# *Oenothera laciniata* Hill Extracts Exhibits Antioxidant Effects and Attenuates Melanogenesis in B16-F10 Cells via Downregulating CREB/MITF/Tyrosinase and Upregulating p-ERK and p-JNK

**DOI:** 10.3390/plants10040727

**Published:** 2021-04-08

**Authors:** Horng-Huey Ko, Yeo-Tzu Chang, Yueh-Hsiung Kuo, Chia-Hsuan Lin, Yih-Fung Chen

**Affiliations:** 1 Department of Fragrance and Cosmetic Science, College of Pharmacy, Kaohsiung Medical University, Kaohsiung 80708, Taiwan; hhko@kmu.edu.tw (H.-H.K.); marian30248@hotmail.com (Y.-T.C.); 2 School of Pharmacy, College of Pharmacy, Kaohsiung Medical University, Kaohsiung 80708, Taiwan; 3 Department of Chinese Pharmaceutical Sciences and Chinese Medicine Resources, China Medical University, Taichung 40402, Taiwan; kuoyh@mail.cmu.edu.tw; 4 Chinese Medicine Research Center, China Medical University, Taichung 40402, Taiwan; 5 Department of Biotechnology, Asia University, Taichung 41354, Taiwan; 6 Graduate Institute of Natural Products, College of Pharmacy, Kaohsiung Medical University, Kaohsiung 80708, Taiwan; fros.ta@hotmail.com; 7 Department of Medical Research, Kaohsiung Medical University Hospital, Kaohsiung 80708, Taiwan

**Keywords:** *Oenothera laciniata*, antioxidant, anti-melanogenesis

## Abstract

*Oenothera laciniata* Hill is a perennial herb traditionally used to alleviate inflammatory complications. This study investigated the antioxidant and anti-melanogenic activities of *O. laciniata*. The methanolic extract (OLM) of *O. laciniata* and its different fractions, including ethyl acetate (OLEF), *n*-butanol (OLBF), and water (OLWF) fractions, were prepared. Antioxidant activities were evaluated by total phenolic content, the radical-scavenging effect on 2,2-diphenyl-1-picrylhydrazyl (DPPH^•^), 2,2′-azino-bis(3-ethylbenzothiazoline-6-sulfonic acid) (ABTS^+•^), and superoxide anion (O2^−•^), reducing capacity, and metal chelating ability. OLM and its fractions exhibited potent antioxidant activity in these in vitro assays, with a correlation between radical-scavenging activity and total phenolic content. OLM and its fractions inhibited the mushroom tyrosinase activity superior to the reference control, ascorbic acid. In B16-F10 melanoma cells, OLM and its fractions significantly decreased melanin production and tyrosinase activity. Mechanistic investigations revealed that OLM and its fractions inhibited tyrosinase and TRP-2 expressions via downregulating MITF and phosphorylated CREB and differentially inducing ERK or JNK phosphorylation. Additionally, OLM and its fractions caused no significant cytotoxicity towards B16-F10 or skin fibroblast cells at concentrations used in these cellular assays. These findings demonstrated the potential of *O. laciniata* extracts as the ideal skin protective agent with dual antioxidant and anti-melanogenic activities.

## 1. Introduction

Melanogenesis is a physiological process by which melanocytes produce melanin pigment and the primary determinant of skin color [1]. Melanin pigment is one of the major defense mechanisms of human skin against deleterious effects of ultraviolet (UV) radiation [2]. Excessive UV exposure usually causes the generation of intracellular reactive oxygen species (ROS) and oxidative stresses, one of the inducing factors for melanin synthesis and skin damage [3,4]. Apart from UV light exposure, various extrinsic and intrinsic factors, such as hormonal changes, inflammation, and age, trigger the biosynthesis of melanin in humans [5,6]. The excessive production and dispersion of melanin leads to skin hyperpigmentation, a common dermatological problem that affects skin appearance, causes aesthetic concerns, and even increases melanoma risk [7]. Therefore, depigmenting agents with additional antioxidant effects that ensure a downregulation of melanogenesis and a decrease in the melanin content are of significant value in cosmetic and pharmaceutical uses.

The biosynthesis of melanin in humans is mediated by tyrosinase and tyrosinase-related proteins (TRPs) TRP-1 and TRP-2 [8,9]. Tyrosinase is the core melanogenic enzyme that catalyzes the first two reactions in melanin synthesis, the formation of 3,4-dihydroxyphenylalanine (l-DOPA) via the hydroxylation of tyrosine and the subsequent formation of dopaquinone through l-DOPA oxidation [9]. Dopaquinone spontaneously converts to dopachrome. The next steps give rise to the synthesis of two types of melanin pigments, pheomelanin and eumelanin, through several oxidation and reduction reactions catalyzed by tyrosinase, TRP-1, and TRP-2 [10]. Hence, the inhibition of tyrosinase or its related proteins TRP-1 and TRP-2 is, therefore, a key tactic for blocking melanin production.

Several transcription factors and signaling pathways are known to regulate melanogenesis [11]. Microphthalmia-associated transcription factor (MITF) is a crucial transcription factor controlling the expression of melanogenic enzymes, such as tyrosinase, TRP-1, and TRP-2 [12]. Many signaling pathways regulate the expression levels of MITF. The phosphorylation of cAMP response element-binding protein (CREB) transcription factor family members is a principal signal that transcriptionally activates MITF expression [13]. Additionally, the mitogen-activated protein kinases (MAPKs) family, including extracellular signal-regulated kinase (ERK), c-Jun N-terminal kinase (JNK), and p38, are potently involved in the melanogenic signaling cascade. It has been demonstrated that the activation of MAPKs contributes to reduced melanin synthesis via MITF degradation and the subsequent downregulation of tyrosinase and its related proteins TRP-1 and TRP-2 [14,15]. Therefore, the downregulation of the transcriptional activity or protein stability of MITF through modulating CREB and MAPK signaling pathways in melanocytes is a potentially valuable strategy to control skin hyperpigmentation.

A wide variety of tyrosinase inhibitors with additional mild to moderate antioxidant activities have been used extensively for hyperpigmentation control, such as hydroquinone, arbutin, kojic acid, and ascorbic acid [16]. Despite their efficacy, many of these agents have been considered unsafe for use in humans [17]. For example, hydroquinone was commonly used for treating hyperpigmentation in the past, however, due to its side effects of dermatitis, edema, allergic reactions, and ochronosis, some countries, including UK, Europe, and Japan, have banned the use of hydroquinone in cosmetics [18]. Kojic acid, an effective and safer depigmenting agent alternative to hydroquinone, also causes side effects, such as dermatitis and erythema, due to cytotoxicity [19]. Many botanicals are considered safer ingredients when applied in cosmetics [20], thus, the development of safe, effective, and innovative depigmenting agents with considerable antioxidant properties from novel natural sources has recently gained much attention.

The genus *Oenothera* (Onagraceae), known as evening primrose, is an annual, biennial, or perennial herbaceous flowering plant native to temperate or subtropical North and South America [21,22]. The *Oenothera* genus is currently distributed in various zones. Five species, including *O. biennis* L., *O. glazioviana* Micheli, *O. laciniata* Hill, *O. stricta* Ledeb. ex Link, and *O. tetraptera* Cav., can be found in Taiwan [21]. Plants of the *Oenothera* genus are known to have a variety of biological activities [23], such as antioxidant [24,25], anti-inflammatory [26,27], anti-microbial [28], anti-viral [29], vasorelaxative [30], anti-aging [31], anti-melanogenic [32], and anti-tumor [33,34] activities. *O. laciniata* has been used as a traditional medicine for inflammatory complications in Taiwan. However, the biological activities and phytoconstituents of this plant are less studied. Recent studies using different study models have found several biological potentials of *O. laciniata* extracts, including antioxidant, anti-inflammatory, anti-melanogenic, and anti-wrinkle effects [35,36,37]. Regarding the phytochemical ingredients of *O. laciniata*, mostly tannins and ellagitannins, including oenotherins A, B, D, F, G, and T_1_, 1,6-di-*O*-galloyl-β-D-glucose, 1,2,3-tri-*O*-galloyl-β-D-glucose, and 1,2,6-tri-*O*-galloyl-β-D-glucose, were isolated from its root, stem, and callus cultures of leaf [38,39,40].

This study investigated the potentials of *O. laciniata* as skin protective agents with dual antioxidant and anti-melanogenic effects by using cell-free and mouse melanoma B16-F10 cell-based assays. The methanolic extract (OLM) of *O. laciniata* and its different fractions, including ethyl acetate (OLEF), *n*-butanol (OLBF), and water (OLWF) fractions, were prepared and tested for total phenolic contents, free radical-scavenging effects on DPPH^•^, ABTS^+•^, and O_2_^−•^, reducing capacity, ferrous chelating effects, as well as the inhibition of mushroom tyrosinase. The cellular safety of OLM and its different fractions was evaluated by the cell viability assay in B16-F10 melanoma cells and Hs68 fibroblast cells. The cellular melanin content and tyrosinase activity in B16-F10 melanoma cells were analyzed to assess the anti-melanogenic effects of OLM and its different fractions. We also examined the underlying anti-melanogenic mechanisms involving melanogenic enzymes, including tyrosinase, TRP-1, and TRP-2, and melanogenic factors, including MITF and phosphorylated CREB, and the signaling of MAPK.

## 2. Results

### 2.1. Total Phenolic Content of Methanolic Extract of O. laciniata (OLM) and Its Different Soluble Fractions

Plant polyphenols, such as flavonoids and phenolic acids, are known to have considerable antioxidant activity by effectively scavenging radicals or inhibiting intracellular ROS production [41]. We, therefore, determined the total phenolic contents of the methanolic extract of *O. laciniata* (OLM) and its different soluble fractions, including ethyl acetate (OLEF), *n*-butanol (OLBF), and water (OLWF) fractions. Total phenolic contents were determined spectrophotometrically according to the Folin–Ciocalteu procedure and calculated as gallic acid equivalents (GAE). Results of total phenolic content determination showed that OLM and its different soluble fractions have a considerable amount of plant phenolics. Among OLM and its fractions, OLBF had the highest total phenolic content (205.2 ± 1.7 mg/g), followed by OLEF (110.9 ± 2.6 mg/g), OLM (107.9 ± 1.6 mg/g), and OLWF (61.1 ± 2.3 mg/g). The phytochemical characterizations of OLM and its different soluble fractions were analyzed by nuclear magnetic resonance (NMR) spectroscopy. Results from the ^1^H-NMR (400 MHZ, MeOH-*d*_4_) analyses indicated that OLM contained complicated mixtures of fatty acids, steroids, phenolic compounds, tannins, and others (Supplementary Figure S1). The liquid–liquid partition led to different compositions between each layer. Fatty acids and steroids were obtained in OLEF (Supplementary Figure S2), phenolic compounds and tannins/sugars were obtained in OLBF (Supplementary Figure S3), and tannins/sugars were obtained in OLWF (Supplementary Figure S4). These results of chemical profiles were consistent with the results of the total phenolic content. Phenolic compounds function as efficient free-radical scavengers by donating hydrogen or electrons and chelating metal ions.

### 2.2. Antioxidant Profile of Methanolic Extract of O. laciniata and Its Different Soluble Fractions

The bioactive agents with dual antioxidant and anti-melanogenic effects are considered beneficial in skin protection and suitable for developing as cosmetic ingredients. We then evaluated the antioxidant abilities of OLM and its different soluble fractions with radical-scavenging assays. Artificial free radicals, such as DPPH^•^, ABTS^+•^, and O_2_^−•^, are commonly used in radical-scavenging assays, providing a reliable antioxidant profile of test samples. The activities of OLM and its fractions to scavenge DPPH^•^, ABTS^+•^, and O_2_^−•^ free radicals were shown in Table 1. The methanolic extract of *O. laciniata* (OLM) and its different soluble fractions exhibited antioxidant activity in all these in vitro tests.

For the DPPH^•^ scavenging activity, OLM and its fractions scavenged DPPH^•^ in a concentration-dependent manner. OLBF was the most active fraction (SC_50_ = 7.2 ± 0.2 μg/mL), with similar potency to the positive control ascorbic acid (SC_50_ = 5.3 ± 0.1 μg/mL). OLM, OLEF, and OLWF had moderate DPPH^•^ radical-scavenging activity, with SC_50_ ranging from 12.5 ± 0.2 to 19.3 ± 1.0 μg/mL. The potency order for DPPH^•^ scavenging activity of OLM and its different soluble fractions was OLBF > OLEF ≈ OLM > OLWF.

For the ABTS^•+^ scavenging activity, OLM and its fractions also showed a concentration-dependent scavenging effect. OLBF was the most potent fraction (SC_50_ = 10.1 ± 0.2 μg/mL), about half as potent as ascorbic acid (SC_50_ = 4.2 ± 0.3 μg/mL). OLM, OLEF, and OLWF had mild to moderate ABTS^+•^ radical-scavenging activity, with SC_50_ ranging from 13.6 ± 0.2 to 33.2 ± 0.6 μg/mL. The potency order for ABTS^+•^ scavenging activity of OLM and its different soluble fractions was OLBF > OLEF ≈ OLM > OLWF.

For O_2_^−•^ scavenging activity, OLEF (SC_50_ = 5.8 ± 0.9 μg/mL) and OLBF (SC_50_ = 6.3 ± 0.2 μg/mL) were the most active fractions, with slightly better potency than ascorbic acid (SC_50_ = 6.5 ± 1.8 μg/mL). OLM and OLWF had mild to moderate ABTS^+•^ radical-scavenging activity, with SC_50_ of 9.1 ± 0.8 and 18.1 ± 0.3 μg/mL, respectively. The potency order for O_2_^−•^ scavenging activity of OLM and its different soluble fractions were OLBF ≈ OLEF > OLM > OLWF.

Overall, the profiles of radical scavenging activities correlated with the results from total phenolic content, showing that OLBF was the most active fraction, followed by OLEF, OLM, and OLWF.

### 2.3. Reducing Capacity and Metal Chelating Activity of Methanolic Extract of O. laciniata and Its Different Soluble Fractions

**Figure 1:**
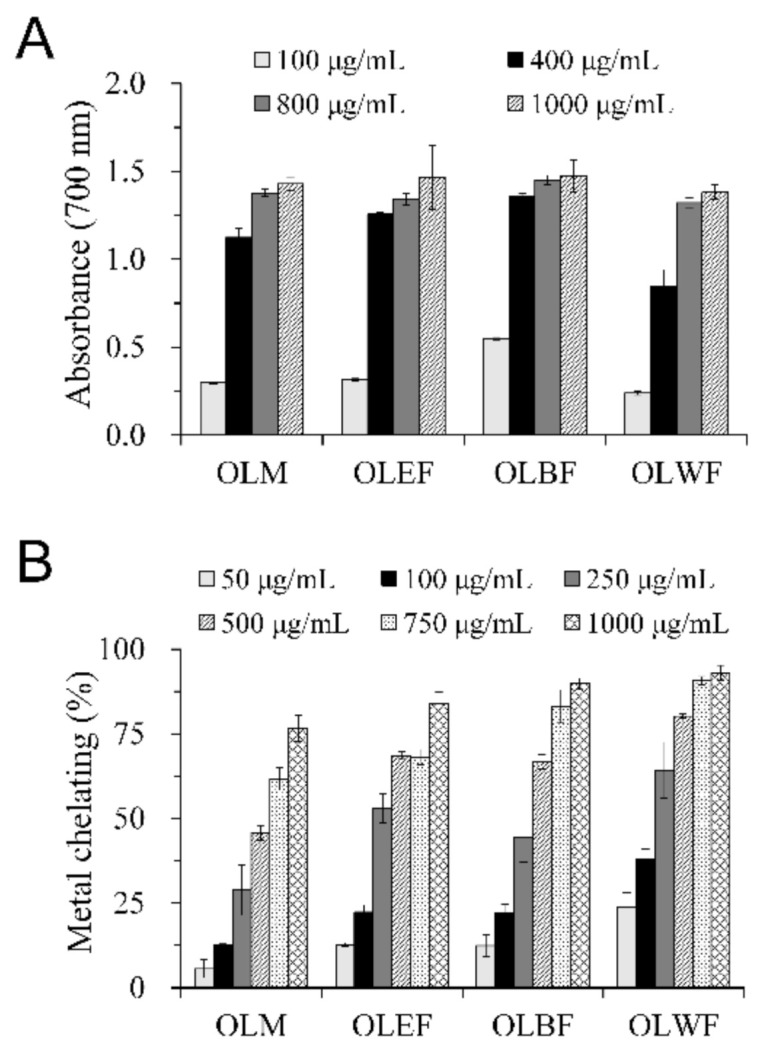
Antioxidant properties of OLM and its different soluble fractions were determined using the ferrous reducing antioxidant capacity assay (**A**) and ferrous-ion chelation assay (**B**). Test samples were the methanolic extract of *O. laciniata* (OLM) and its different soluble partition fractions, including ethyl acetate (OLEF), *n*-butanol (OLBF), and water (OLWF). (**A**) The reducing power was determined using the Fe^3+^–Fe^2+^ system with higher absorbance at 700 nm, indicating a stronger reducing power. (**B**) The Fe^2+^ chelating activity (%) was calculated based on the absorbance of reaction mixtures measured at 562 nm. Values are the mean ± SD of independent triplicate experiments.

The reducing capacity and the ferrous ion chelating activities of OLM and its different soluble fractions were also evaluated. The reducing capacity of an agent is considered as a significant indicator of antioxidant activity. Metal chelating activity contributes to one of the antioxidant mechanisms by obstructing the activity of catalytic metals present in the surrounding environment. Figure 1A showed the ferric reducing antioxidant capacity of OLM and its different soluble fractions determined using the Fe^3+^–Fe^2+^ system, in which higher absorbance indicating a more substantial reducing power. The reducing capacities of OLM and its fractions were elevated with the increase in concentration. Based on results of 400 μg/mL, the order of the reducing capacity was OLBF > OLEF > OLM > OLWF. Figure 1B showed the ferrous ion chelating activities of OLM and its different soluble fractions, indicating that Fe^2+^ was chelated in a concentration-dependent manner. Based on results of 500 μg/mL, the potency order for the metal chelating activity of OLM and its different soluble fractions was OLWF > OLBF ≈ OLEF > OLM.

### 2.4. Mushroom Tyrosinase Inhibitory Activity of Methanolic Extract of O. laciniata and Its Different Soluble Fractions

Tyrosinase is the key enzyme that catalyzes the first and rate-limiting step of melanin biosynthesis in living organisms, and the down-regulation of its activity effectively inhibits melanogenesis. The mushroom tyrosinase (EC 1.14.18.1) inhibitory assay, a common in vitro screening platform of the depigmenting agent, was performed to evaluate the anti-melanogenic potentials of OLM and its different soluble fractions. The concentrations of OLM and its fractions that inhibited 50% of the mushroom tyrosinase activity (IC_50_) were shown in Table 1. Ascorbic acid was used as a positive control in the mushroom tyrosinase inhibition assay (IC_50_ = 162.4 ± 11.3 μg/mL). Notably, OLM and its different soluble fractions exhibited potent tyrosinase inhibitory activities, with IC_50_ ranging from 80.5 ± 0.9 to 106.8 ± 2.7 μg/mL. Among different fractions, OLWF (IC_50_ = 80.5 ± 0.9 μg/mL) and OLBF (IC_50_ = 84.8 ± 0.6 μg/mL) were the most active ones, with about twofold of inhibitory effects than ascorbic acid (IC_50_ = 162.4 ± 11.3 μg/mL). These results demonstrated that OLM and its different soluble fractions possessed anti-tyrosinase activity, and the rank order of efficacy inhibiting mushroom tyrosinase activity was OLWF ≈ OLBF > OLM > OLEF.

### 2.5. Effect of Methanolic Extract of O. laciniata and Its Different Soluble Fractions on Cell Viability of B16-F10 Melanoma and Hs68 Skin Fibroblast Cells

**Figure 2:**
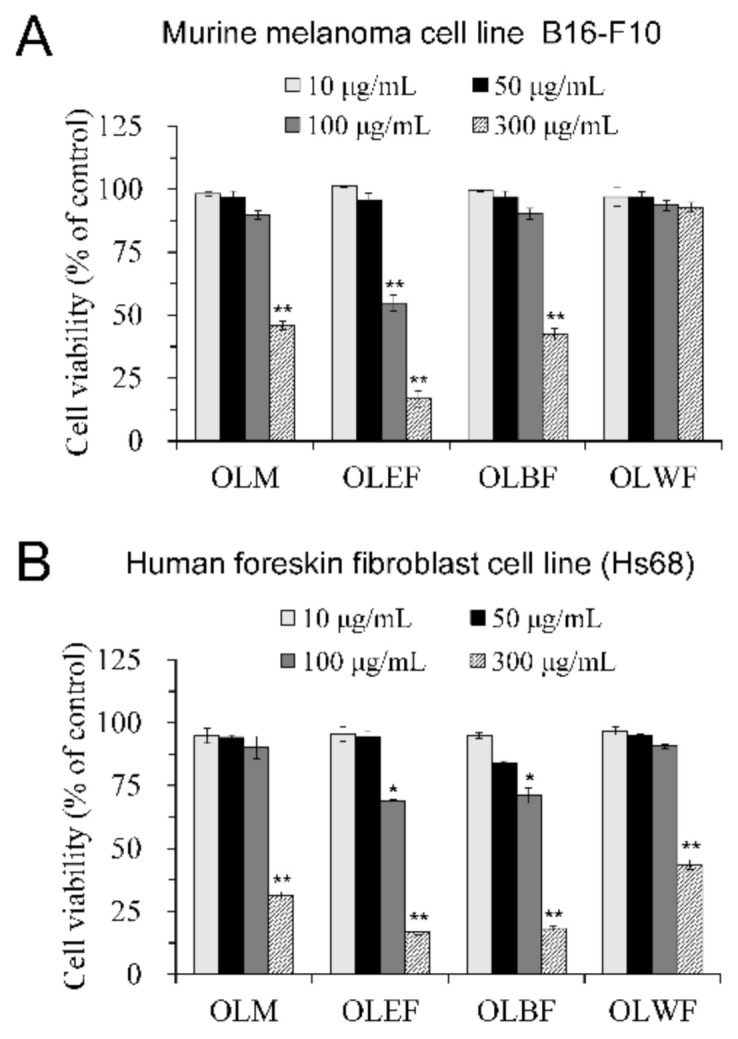
Effects of OLM and its different soluble fractions on the cell viability of B16-F10 melanoma (**A**) and Hs68 fibroblast cells (**B**). Cells were treated with vehicle control or 10–300 μg/mL of OLM and its fractions, including ethyl acetate (OLEF), *n*-butanol (OLBF), and water (OLWF), for 48 h, with an XTT assay used to quantify their effects on cell viability. The cell viability was calculated as a percentage of the viability in control. Values are the mean ± SD of three independent experiments. * *p* < 0.05, ** *p* < 0.01, compared with the control group.

The non-cytotoxic concentrations of OLM and its different soluble fractions were determined before evaluating their effects on cellular tyrosinase activity, melanin content, and melanogenic pathways. Results from cell viability assays on B16-F10 melanoma cells and Hs68 fibroblast cells demonstrated that OLM and its fractions had no cytotoxic effects at concentrations under 50 μg/mL, with cell viability remained higher than 90% after treatment for 48 h (Figure 2A,B). At the concentration of 100 μg/mL, OLM and OLWF did not affect the cell viability, but OLEF and OLBF caused deleterious effects on B16-F10 melanoma cells and Hs68 fibroblast cells (Figure 2A,B). At the concentration of 300 μg/mL, OLWF did not affect the viability of B16-F10 melanoma cells but significantly reduced the cell viability of Hs68 fibroblast cells to lower than 50% (Figure 2A,B). Moreover, OLM, OLEF, and OLBF reduced cell viability of B16-F10 melanoma cells to lower than 50% at the concentration of 300 μg/mL. Therefore, the following cellular assays on the anti-melanogenic effect and the underlying molecular mechanisms were performed at a concentration lower than 50 μg/mL.

### 2.6. Anti-Melanogenic Effects of Methanolic Extract of O. laciniata and Its Different Soluble Fractions in B16-F10 Melanoma Cells: Cellular Melanin Content and Tyrosinase Activity

**Figure 3:**
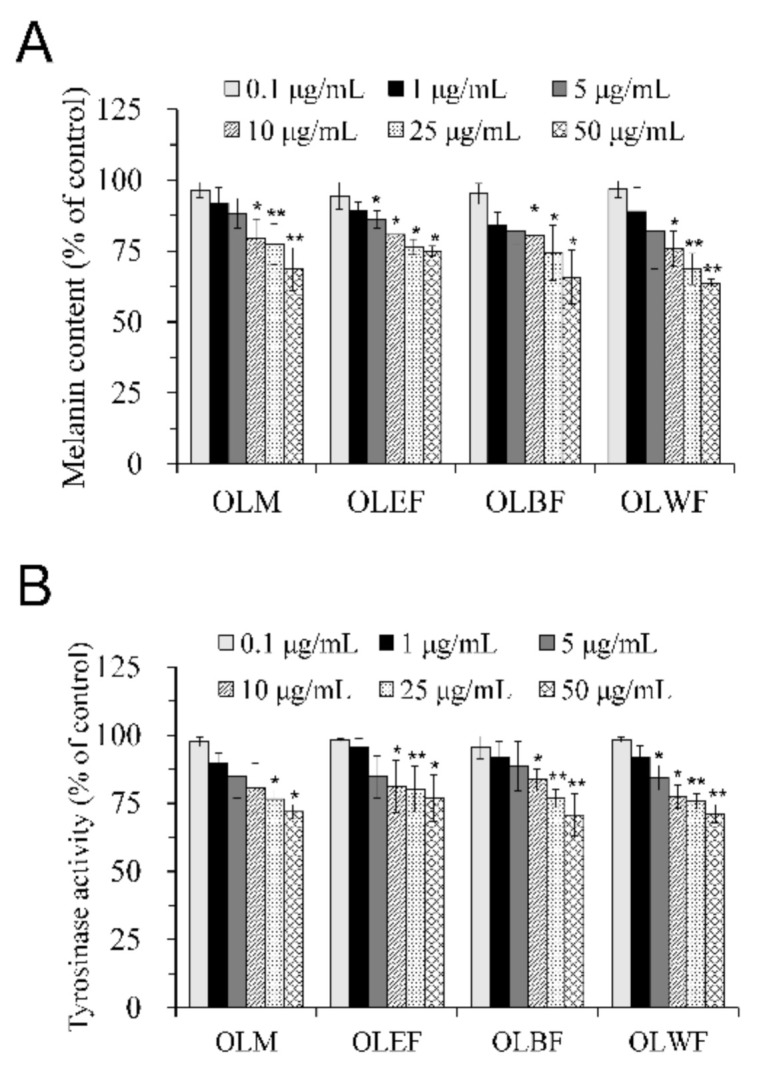
Inhibitory effects of OLM and its different soluble fractions on the cellular melanin production (**A**) and tyrosinase activity (**B**) in B16-F10 cells. Melanin production and tyrosinase activity in B16-F10 cells treated with vehicle control or non-cytotoxic concentrations (0.1–50 μg/mL) of OLM and its fractions for 48 h were measured. The melanin content and tyrosinase activity were represented as a percentage of the content in control. Values are the mean ± SD of three independent experiments. * *p* < 0.05, ** *p* < 0.01, compared with control group.

A good depigmenting agent not only effectively inhibits cellular tyrosinase activity but also decreases melanin content. We next applied cell-based assays on B16-F10 melanoma cells to determine the inhibitory effect of OLM and its different soluble fractions on cellular melanin content and tyrosinase activity. B16-F10 melanoma cells were treated with test samples for 48 h at concentrations up to 50 μg/mL, known to be non-toxic. Results from cellular melanin content assays demonstrated that OLM and its fractions at concentrations ranging from 10 to 50 μg/mL significantly reduced cellular melanin production in a concentration-dependent manner (Figure 3A). Consistently, OLM and its fractions at concentrations ranging from 10 to 50 μg/mL significantly inhibited cellular tyrosinase activities in a concentration-dependent manner (Figure 3B). These results suggested that the decrease in cellular melanin in B16-F10 cells treated with non-cytotoxic concentrations of OLM and its fractions might be due to the inhibition of tyrosinase activities.

### 2.7. Anti-Melanogenic Signaling of Methanolic Extract of O. laciniata and Its Different Soluble Fractions in B16-F10 Melanoma Cells: P-CREB-MITF-Tyrosinase Pathway and Phosphorylations of ERK and JNK

**Figure 4:**
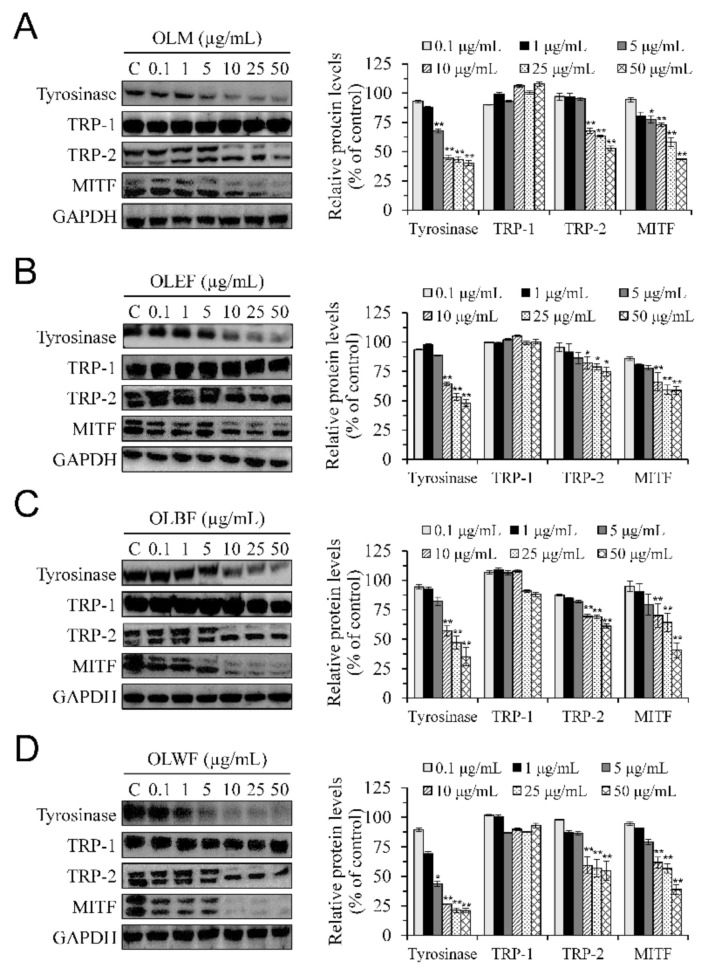
The effects of OLM (**A**) and its fractions, including OLEF (**B**), OLBF (**C**), and OLWF (**D**), on protein expressions of tyrosinase, TRP-1, TRP-2, and MITF in B16-F10 cells. B16-F10 melanoma cells were treated with test samples for 48 h at concentrations up to 50 μg/mL, known to be non-toxic. Left panels, representative immunoblots. Right panels, densitometry analyses of the relative ratio of protein/GAPDH protein, represented as percentages of the control group. Columns, mean ± SD of three independent experiments. * *p* < 0.05, ** *p* < 0.01, compared with control group.

During melanogenesis, the transcription factor MITF is a crucial regulator that promotes the expression of melanogenic enzymes, such as tyrosinase, TRP-1, and TRP-2 [12]. To investigate the molecular mechanisms by which OLM and its fractions inhibit melanogenesis, we examined the changes in expression of these key melanogenic proteins, including tyrosinase, TRP-1, TRP-2, and MITF. Results from western blotting analyses indicated that treatment of OLM and its fractions at concentrations ranging from 10 to 50 μg/mL for 48 h caused a concentration-dependent reduction in protein levels of tyrosinase, TRP-2, and MITF, but not in that of TRP-1 (Figure 4A–D). These results suggested that the anti-melanogenic effects of OLM and its fractions in B16-F10 cells involve the decrease in tyrosinase and TRP-2 expressions via the downregulation of MITF.

**Figure 5:**
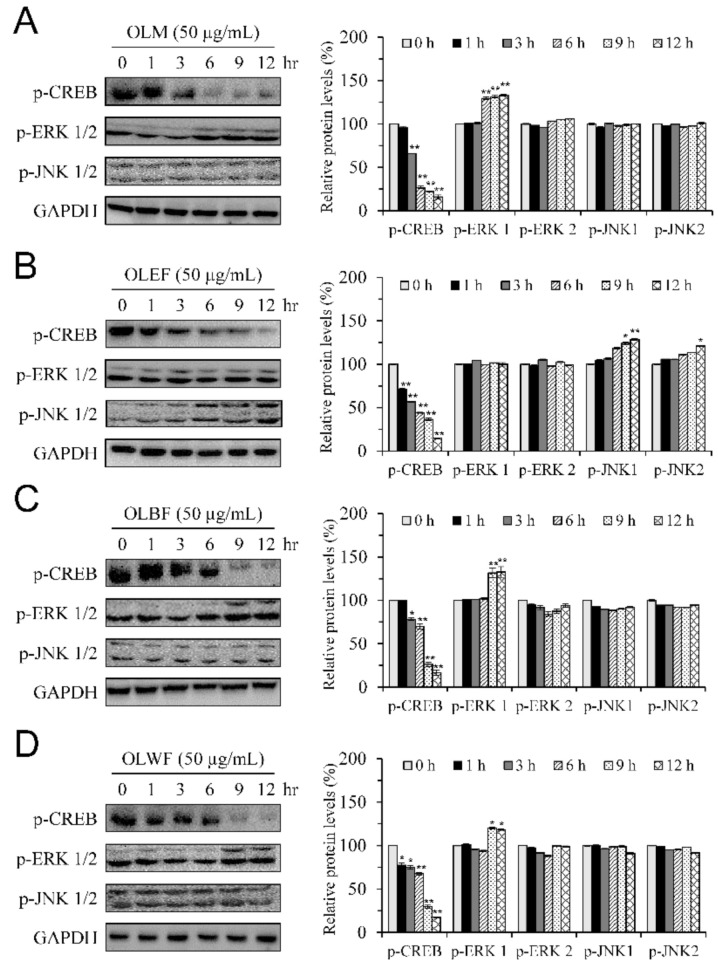
Effects of OLM (**A**) and its fractions, OLEF (**B**), OLBF (**C**), and OLWF (**D**), on the phosphorylation levels of CREB, ERK1/2, and JNK1/2 in B16-F10 cells. B16-F10 melanoma cells were treated with 50 μg/mL of test samples for for 1, 2, 6, 9, and 12 h: left panels are representative immunoblots. Right panels are densitometry analyses of the relative ratio of phosphorylated-protein (p-protein)/GAPDH protein, represented as percentages of the control group. Columns, mean ± SD of three independent experiments. * *p* < 0.05, ** *p* < 0.01, compared with control group.

During melanogenesis, the phosphorylation of CREB is known to induce the expression of MITF, leading to the upregulation of tyrosinase expression and subsequent melanin synthesis [11]. Moreover, the activation of ERK and JNK is associated with the degradation of MITF, inhibition of tyrosinase synthesis, and reduction of melanin production [14,15]. We next determined the effects of OLM and its fractions on the activation of CREB, ERK, and JNK via detecting their phosphorylation levels. The time-course experiment with the treatment of OLM and its fractions at a concentration of 50 μg/mL for 1, 2, 6, 9, and 12 h was conducted. Treatment with OLM and its fractions for 3 to 12 h caused a time-dependent decrease in the phosphorylation of CREB (Figure 5A–D). The levels of phosphorylated ERK1 were significantly increased upon the treatment with OLM and its fractions OLBF and OLWF (Figure 5A,C,D), but not with OLBF (Figure 5B), for 6 to 9 h. In contrast, OLM and its fractions OLBF and OLWF did not cause significant alterations on the phosphorylation levels of ERK2 and JNK1/2 (Figure 5A,C,D). OLEF significantly increased the phosphorylation of JNK1 at 6 and 9 h and that of JNK2 at 9 h (Figure 5B). These results suggested that the inhibitory effects of OLM and its fractions on the expression of tyrosinase-related proteins and melanin production, at least partially through the downregulation of MITF expression and CREB phosphorylation, as well as the differential induction of ERK or JNK phosphorylation. Anti-melanogenic effects of OLM, OLBF, and OLWF involved an increase in ERK phosphorylation, whereas that of OLEF involved an upregulation of JNK phosphorylation.

## 3. Discussion

*O. laciniata* is a perennial herbaceous plant that is traditionally used to alleviate inflammatory complications. The knowledge of biological activities and phytoconstituents of *O. laciniata* has been limited until recent studies revealing its anti-inflammatory and skin protective potentials. The dichloromethane fraction of *O. laciniata* extract was demonstrated to possess the anti-inflammatory activity in lipopolysaccharide-induced RAW 264.7 macrophages via inhibiting the production of nitric oxide, prostaglandin E_2_, and pro-inflammatory cytokines [35]. A study in human dermal fibroblasts demonstrated that the methanolic extract of *O. laciniata* promoted procollagen production and inhibited the expression or activities of MMPs, indicating the anti-wrinkle potentials of *O. laciniata* [36]. The antioxidant and depigmenting effects of *O. laciniata* were examined recently [37]. Kim et al. found that the methanolic extract of *O. laciniata* decreased intracellular tyrosinase activity and down-regulated mRNA expressions of melanogenic factors, including tyrosinase, TRP-1, TRP-2, and MITF-M, of mouse melanocyte melan-a cells [37]. However, the direct effects on tyrosinase and the detailed mechanisms underlying melanogenesis inhibition by *O. laciniata* were not thoroughly investigated. In an attempt to develop an effective skin protective agent, this study investigated the dual antioxidant and anti-tyrosinase potentials of the methanolic extract (OLM) of *O. laciniata* and its different fractions, including ethyl acetate (OLEF), *n*-butanol (OLBF), and water (OLWF) fractions. Using various in vitro assays, we found that OLM and its different fractions exhibited potent antioxidant and direct anti-tyrosinase effects. With the cell model of mouse melanoma B16-F10 cells, we also revealed the anti-melanogenic capabilities of OLM and its different fractions by mechanisms involving the decrease in tyrosinase and TRP-2 expressions and melanin productions via downregulating MITF and p-CREB and differentially upregulating p-ERK or p-JNK. A practical consideration would be that most solvents used in this study, except water, were not suitable for cosmetic application because of their possible toxicity. However, the amount of residual solvents remained in the samples after processes of concentration at reduced pressure and crystallization could be very limited and even undetectable. Alternative extraction approaches such as green or solvent-free extraction techniques are worth applying for the further development of *O. laciniata* as the cosmeceutical agent for hyperpigmentation control.

The exploration of agents capable of controlling hyperpigmentation is a trending research topic in health and cosmetics. UV overexposure leads to hyperpigmentation by inducing the overproduction of ROS [42]. Downregulation of the expressions or activities of tyrosinase that determines the initial rate of melanin biosynthesis is effective for inhibiting melanogenesis. Therefore, the development of antioxidants, or free radical scavengers, and tyrosinase inhibitors constitute critical approaches for reducing excessive skin pigmentation. The antioxidants derived from natural products have gained much attention due to their relative safety compared to synthetic ones [43]. The pursuit of effective depigmenting agents with fewer side effects has prompted investigations on developing natural products with dual antioxidant and anti-tyrosinase activities. Plant polyphenols have been shown to act as antioxidants because of their redox properties, allowing them to act as reducing agents, hydrogen donors, single-oxygen quenchers, or metal chelators [44,45]. Additionally, several plant phenolic compounds have been identified as having anti-melanogenic properties via tyrosinase inhibition [46,47,48]. In the present study, we evaluated the antioxidant profiles of OLM and its different fractions by employing multiple analyses, including the radical-scavenging effect on DPPH^•^, ABTS^+•^, and O_2_^−•^, ferric reducing power, and metal-chelating capacity. We found a dose-dependent increase in antioxidant potentials of OLM and its different fractions in all the analytical studies. Our analyses on total phenolic content determination showed a considerable amount of plant phenolics in OLM and its different soluble fractions, correlating with their radical-scavenging activity and ferric reducing capability. Our results from mushroom tyrosinase inhibition assay showed that OLM and its different fractions exhibited a direct inhibition of tyrosinase, to an extent even more potent than ascorbic acid. Such direct inhibition of tyrosinase activity of OLM and its different fractions is, at least partially, contributed by the considerable amounts of phenolic ingredients and remarkable antioxidant effects. Therefore, we deduce that OLM and its different fractions may help diminish the oxidative damage caused by free radicals, thus reducing hyperpigmentation. The previous investigation also indicated that the degree of inhibition of melanogenesis by some plant extracts, for example, *Phoenix dactylifera*, is correlated with its total phenolic content [49].

Apart from the inhibition on the activity of key melanogenic enzymes tyrosinase and its related proteins TRP-1 and TRP-2, skin pigmentation can be suppressed via modulating melanogenesis transcriptional factors, such as CREB and MITF. The CREB/MITF pathway is known to play a significant role in regulating melanin biosynthesis in melanocytes. It has been demonstrated that that CREB phosphorylation is a major signal for the enhanced expression of MITF, which increases melanin production by activating the transcription of tyrosinase, TRP-1, and TRP-2 [12,13]. Therefore, the downregulation of the CREB/MITF pathway is a practical strategy for the effective inhibition of melanogenesis. Our results demonstrated that OLM and its different fractions concentration-dependently suppressed MITF expressions of B16-F10 cells, accompanied by the decreased expressions of its downstream melanogenic enzymes tyrosinase and TRP-2. Consistently, the phosphorylation levels of CREB were reduced by treatment with OLM and its different fractions in a dose-dependent manner. Thus, the suppression of melanogenic enzymes and melanin production by OLM and its different fractions involve the inhibition of CREB phosphorylation and MITF expressions. Similar effects were found in previous investigations into the anti-melanogenic natural products, such as *Phyllostachys nigra* var. henonis [50], *Phyla nodiflora* [51], and rottlerin [52].

In addition to CREB signaling, the MAPK family, including ERK, JNK, and p38, are known to have crucial roles in regulating melanogenesis. It has been shown that the activation of MAPK signaling cascades causes MITF phosphorylation at serine-73, which leads to the subsequent ubiquitination and proteasomal degradation of MITF, finally diminishing tyrosinase synthesis and melanin production [14,15,53]. Therefore, the downregulated MITF expressions and stability through modulating MAPK signaling in melanocytes are promising targets for the development of cosmeceuticals or therapeutics for skin hyperpigmentation. The current investigation is the first to report the effect of *O. laciniata* extracts on the MAPK family of ERK and JNK that regulates MITF stability and melanin productions. Our results showed that OLM and its different fractions at non-cytotoxic concentrations effectively activated the phosphorylation of ERK-1 in a time-dependent manner. Regarding the effects on JNK phosphorylation, OLEF time-dependently increased the phosphorylation of JNK1 and JNK2. Moreover, such increases in ERK and JNK phosphorylation were accompanied by a decrease in CREB phosphorylation. These effects triggered by OLM and its different fractions may lead to MITF downregulation and reduced synthesis of melanogenic enzymes, thereby inhibiting melanogenesis. Previous investigations also demonstrated that effective hypopigmenting natural products could downregulate MITF protein expression and inhibit tyrosinase-related protein synthesis and melanin production through promoted activation of MAP kinase family of ERK [51,54,55,56,57], JNK [51,57,58,59], or p38 [51,56,57].

## 4. Materials and Methods

### 4.1. Chemicals and Reagents

2,2′-azino-bis(3-ethylbenzothiazoline-6-sulfonic acid) diammonium salt (ABTS), 2,2-diphenyl-1-picrylhydrazyl (DPPH), xanthine, xanthine oxidase (from buttermilk, 0.07 U/mg), ethylenediaminetetracetic acid disodium salt dehydrate (EDTA-2Na), nitrotetrazolium blue chloride (NBT), trichloroacetic acid (TCA), 3-(2-pyridyl)-5,6-diphenyl-1,2,4-triazine-4′,4″-disulfonic acid sodium salt (ferrozine), iron chloride hexahydrate (FeCl_3_), phenazine methosulfate (PMS), 2,3-bis(2-methoxy-4-nitro-5-sulfophenyl)-2*H*-tetrazolium-5-carboxanilide inner salt (XTT), l-tyrosine, mushroom tyrosinase (EC 1.14.18.1), l-DOPA, and cell culture reagents were all purchased from Sigma-Aldrich (St. Louis, MO, USA). Sodium hydroxide (NaOH) and sodium carbonate (Na_2_CO_3_) were obtained from Shimakyu’s Pure Chemicals (Osaka, Japan). Ferrous chloride (FeCl_2_) was obtained from Afla-Aesar (Heysham, UK). Folin-Ciocalteu’s phenol reagent (FC reagent) was purchased from E. Merck (Darmstadt, Germany). L-Ascorbic acid, potassium persulfate (K_2_S_2_O_8_), and potassium hexacyanoferrate (III)(K_3_Fe(CN)_6_) were purchased from J. T. Baker (Austin, TX, USA). Antibodies for tyrosinase, TRP-1, TRP-2, MITF, p-CREB (Ser133), GAPDH, and goat anti-mouse and anti-rabbit horseradish peroxidase-conjugated immunoglobulin G (IgG) were purchased from Santa Cruz Biotechnology (Santa Cruz, CA, USA). Antibodies for phospho-ERK (p-ERK; Thr202/Tyr204) and p-JNK (Thr183/Tyr185) were purchased from Cell Signaling Technology (Danvers, MA, USA). Enhanced chemiluminescence reagent and nitrocellulose blotting membranes membrane were obtained from Millipore (Billerica, MA, USA). All materials for SDS-PAGE were obtained from Bio-Rad (Hercules, CA, USA).

### 4.2. Plant Materials and Preparation of Oenothera laciniata Extracts

**Figure 6:**
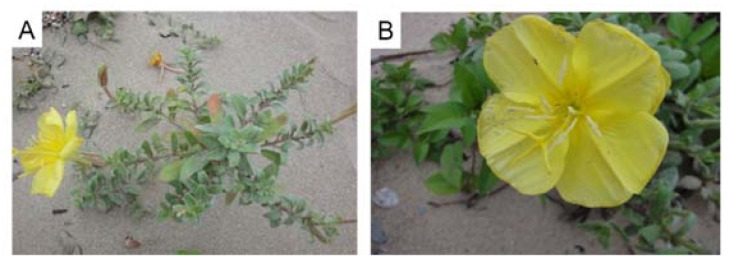
The pictures of *O. laciniata,* showing the aerial part (**A**) and the flower (**B**).

*O. laciniata* (Figure 6) was collected in July 2010 from Tanghoudao Beach, Beigan Township, Lienchiang County (the Matsu Islands), Taiwan, and authenticated by Mr. Sin-Yang, a senior researcher of the Biological Resources Research Center in Matsu, Taiwan. A voucher specimen (2010-OL-Klab) has been deposited in the Department of Fragrance and Cosmetic Science, Kaohsiung Medical University, Kaohsiung, Taiwan. The whole plants were cleaned, dried at room temperature for two weeks, and cut into pieces for extraction. The materials (3.89 kg) were extracted with MeOH at room temperature for three days and with two additional three-day extractions of the material under the same condition. The crude extracts were filtered and concentrated under reduced pressure to yield the methanolic extract (OLM, 378.6 g). The dried OLM (378.6 g) was dissolved in H_2_O and further fractionated with EtOAc, *n*-BuOH, and H_2_O to yield an EtOAc-soluble fraction (OLEF, 94.0 g), an *n*-BuOH-soluble fraction (OLBF, 102.9 g), and an H_2_O-soluble fraction (OLWF, 120.3 g), respectively.

### 4.3. Total Phenolic Content Assay

The total polyphenol content of samples was measured using the Folin–Denis assay [60]. Gallic acid was used to create a calibration curve for determining the total phenolic content in each sample. The assay mixture in each well of the 96-well plates contained 0.2 N Folin-Ciocalteu’s reagent (100 μL) and different concentrations of test samples (20 μL). The solutions were rapidly mixed and allowed to stand for 1 min. After adding 80 μL of 7% Na_2_CO_3_, the mixture was mixed and allowed to stand for 30 min. The absorbance was measured at 725 nm using a microplate reader (μQuant™, BioTek, Winooski, VT, USA). The polyphenol content was quantified using a standard curve prepared using gallic acid (GAE) and represented as mg GAE/g. All determinations were performed in independent triplicate.

### 4.4. DPPH Radical (DPPH^•^) Scavenging Assay

The DPPH radical scavenging assay was performed according to the methods reported previously [61], with slight modifications. In brief, 150 μL (0.1 mM) of DPPH^•^ methanolic solution was mixed with 50 μL of various concentrations of the test sample, or positive control (ascorbic acid) dissolving in methanol. The mixture was then vortexed vigorously and left for 30 min in the dark. The absorbance was measured at 517 nm using a microplate reader (μQuant™, BioTek, Winooski, VT, USA). The DPPH^•^-scavenging activity was calculated using the following formula, DPPH^•^-scavenging activity (%) = [1 − (S − SB)/(C − CB)] × 100, where S, SB, C, and CB are the absorbance of the sample, the blank sample, the control, and the blank control, respectively.

### 4.5. ABTS Cation Radical (ABTS^+•^) Scavenging Assay

The ABTS^+•^ scavenging capacity was measured according to the methods reported previously [62]. Briefly, a mixture containing 170 μL of ABTS^+•^ and 30 μL of test samples, negative control (PBS; phosphate-buffered saline), or positive control (ascorbic acid) were thoroughly mixed and incubated for 7 min at room temperature, then measured the absorbance at 734 nm using a microplate reader (μQuant™, BioTek, Winooski, VT, USA). The percentage of ABTS cation free radical scavenging activity was calculated using the same formula as for the DPPH free radical scavenging assay.

### 4.6. Superoxide Anion Radical (O_2_^−•^) Scavenging Assay

The procedures of O_2_^−•^ scavenging activity were performed according to the methods reported previously [63]. In the O_2_^−•^ scavenging assay, xanthine (0.1 mM, 15 μL) in 50 mM phosphate buffer (pH 7.4), NBT (0.25 mM, 16 μL), EDTA (0.1 mM, 14 μL), and 100 μL of various concentrations of test compounds were mixed in the 96-well plates. Reactions were initiated by adding 0.5 U xanthine oxidase. After 30 min, the absorbance of each mixture was measured at 540 nm using a microplate reader (μQuant™, BioTek, Winooski, VT, USA). The percentage of superoxide anion scavenging activity was calculated using the same formula as for the DPPH free radical scavenging assay.

### 4.7. Ferric Reducing Antioxidant Capacity Assay

The ferric reducing antioxidant capacity was measured according to the previously reported methods [64], with slight modifications. Briefly, a mixture containing 100 μL of test samples, 250 μL of phosphate buffer (0.2 M, pH 6.8), and 250 μL of 1% potassium ferricyanide [K_3_Fe(CN)_6_] were thoroughly mixed. After incubation at 50 °C for 20 min, 250 μL of 10% trichloroacetic acid (TCA) was added. After incubation at room temperature for 10 min, 90 μL of the above mixture solution was mixed with 20 μL of 0.1% ferric chloride (FeCl_3_) in the 96-well plate. The formation of Perl’s Prussian blue was measured at 700 nm using a microplate reader (μQuant™, BioTek Winooski, VT, USA). Increased absorbance of the reaction mixture indicates increased reducing capacity.

### 4.8. Ferrous Iron (Fe^2+^) Chelating Assay

The Fe^2+^ metal chelating activity was measured according to the previously reported methods [65], with slight modifications. In this assay, the formation of the ferrozine-Fe^2+^ complex is interfered with by the existence of chelating agents, resulting in quantitatively fading of the red color. Briefly, a mixture containing 10 μL FeCl_2_ (1 mM), 120 μL methanol, and 50 μL of various concentrations of test compounds were mixed and incubated for 10 min at room temperature in the 96-well plates. Reactions were initiated by adding 10 μL ferrozine (2.5 mM). After 10 min, the absorbance of each mixture was measured at 562 nm using a microplate reader (μQuant™, BioTek, Winooski, VT, USA). The Fe^2+^ chelating activity was using the same formula as for the DPPH free radical scavenging assay.

### 4.9. Mushroom Tyrosinase Inhibition Assay

The tyrosinase inhibitory assay was performed using l-tyrosine as a substrate according to the previously reported method [61]. Briefly, test samples or positive control (ascorbic acid) were dissolved in DMSO/methanol before diluted by phosphate buffer (pH 6.8). The assay was performed in a 96-well plate containing 100 μL of test samples or ascorbic acid, 20 μL of mushroom tyrosinase (1000 U/mL), and 80 μL l-tyrosine (2.0 mM). The absorbance of the mixture was measured at 490 nm using a microplate reader (μQuant™, BioTek Winooski, VT, USA) after 30 min of incubation at room temperature. The percentage inhibition of mushroom tyrosinase was calculated according to the following equation: Inhibition (%) = [1 − (S − SB)/(C − CB)] × 100, where S, SB, C, and CB are the absorbance of the sample, the blank sample, the control, and the blank control, respectively.

### 4.10. Cell Culture

The mouse melanoma cell line B16-F10 (BCRC 60031) and human foreskin fibroblast cell line Hs68 (BCRC 60038) were purchased from the Bioresource Collection and Research Center (BCRC), Hsinchu, Taiwan. Cells were cultured in Dulbecco’s Modified Eagle Medium (DMEM) containing 10% fetal bovine serum (FBS), 4 mM L-glutamine, 100 U/mL penicillin, 100 μg/mL streptomycin, and 0.25 μg/mL amphotericin B. Cells were maintained at 37 °C in a humidified incubator with 5% CO_2_ and 95% air. The cell culture medium was replaced every 2–3 days, and confluent cells were passed every 3–5 days with trypsinization.

### 4.11. Cell Viability Assay

Cell viability was determined by using the 2,3-bis-(2-methoxy-4-nitro-5-sulfophenyl)-2H-tetrazolium-5-carboxanilide (XTT) assay. Mitochondrial dehydrogenases metabolize XTT to an orange formazan dye. Briefly, cells were plated at a density of 1 × 10^4^ cells/well in 96-well plates for 24 h, and the treatments were applied for 48 h with different concentrations of the samples. For XTT assay, after the treatment of the cells, the culture medium was removed. Cells were then incubated with XTT solution for 4 h at 37 °C in a humidified atmosphere of 5% CO_2_. The absorbance at 492 nm was measured using a microplate reader (μQuant™, BioTek, Winooski, VT, USA). Cell viability was calculated as follows: Cell viability (%) = (absorbance of the tested cells/absorbance of the control) × 100.

### 4.12. Cellular Melanin Content and Tyrosinase Activity Assays

Cellular melanin content and tyrosinase activity were determined as described previously [46]. Briefly, B16-F10 cells were plated at a density of seeded 1 × 10^5^ cells/well into 24-well plates for 24 h. Treatments were applied with the indicated concentrations for an additional 48 h. Cells were then harvested, washed twice with PBS, and centrifuged at 12,000 rpm for 10 min. For cellular melanin contents, cell pellets were then suspended in 2.0 N NaOH (100 μL) and incubated at 80 °C for 1 h to solubilize melanin. The absorbance at 405 nm was measured using a microplate reader (μQuant™, BioTek, Winooski, VT, USA). Cellular melanin content was calculated as follows: Melanin content (%) = (absorbance of the tested cells/absorbance of the control) × 100. For tyrosinase activity, treated cells were solubilized with PBS containing 1% Triton X-100 prior to centrifugation. Then, 100 μL of supernatants were mixed with L-DOPA (100 μL, 1 mg/mL) for 3 h at 37 °C. Absorbance at 490 nm was measured using a microplate reader (μQuant™, BioTek, Winooski, VT, USA). Tyrosinase activity was calculated as follows: Tyrosinase activity (%) = (absorbance of the tested cells/absorbance of the control) × 100.

### 4.13. Western Blotting Analyses

B16-F10 cells were treated with test samples in different concentrations to determine levels of melanogenic proteins, such as tyrosinase, TRP-1, TRP-2, and MITF. Besides, B16-F10 cells were treated with 50 μg/mL of test samples for indicated time to determine melanogenesis signaling pathways of p-ERK, p-JNK, and p-CREB. Cells were harvested with ice-cold modified radioimmune precipitation assay buffer, containing a protease inhibitor mixture (Sigma-Aldrich, St. Louis, MO, USA), 50 mM Tris, 150 mM NaCl, 1% NP40, 0.1% sodium dodecyl sulfate, 0.5% sodium deoxycholate, 10 mM phenylmethylsulfonyl fluoride, and 1 mM sodium orthovanadate. Cell lysates were centrifuged at 15,000 × *g* for 30 min at 4 °C, and the supernatant containing proteins was collected. Protein concentrations were determined by using the Bio-Rad protein assay kit. Equal amounts of protein were separated on the polyacrylamide gel and transferred to nitrocellulose blotting membranes. Membranes were blocked, incubated with the indicated primary antibody, washed, and incubated with the corresponding horseradish peroxidase-conjugated secondary antibodies. Protein bands were visualized with the enhanced chemiluminescence reagent with the AlphaImager HP system (Alpha Innotech, USA). Bands in the immunoblots were quantified using the ImageJ software (National Institutes of Health, Bethesda, MD, USA).

### 4.14. Statistical Analyses

All data are presented as the mean ± standard deviation (SD) values derived from independent triplicate experiments. The controls and treatment groups were compared using Student’s *t*-test (SPSS 13 Inc., Chicago, IL, USA). Differences between groups were determined to be significant when *p* < 0.05.

## 5. Conclusions

The current investigation determined the antioxidant and anti-tyrosinase potentials of *O. laciniata*, as well as the signaling pathway involved in anti-melanogenic mechanisms. The methanolic extract of *O. laciniata* (OLM) and its different soluble fractions, including ethyl acetate (OLEF), *n*-butanol (OLBF), and water (OLWF) fractions, were used to develop a safe skin protective agent with antioxidant and depigmenting effects. With various in vitro assays, we demonstrated that OLM and its different fractions possessed significant antioxidant activities attributable to the total phenolic contents, radical scavenging activities, and metal chelating ability. Results from mushroom tyrosinase inhibition assay showed that OLM and its different fractions exhibited substantial anti-tyrosinase activities. Consistent with the inhibition on mushroom tyrosinase, OLM and its different fractions markedly decreased cellular tyrosinase activity and cellular melanin content in B16-F10 melanoma cells. Our mechanistic investigations in B16-F10 cells revealed that the anti-melanogenic effects by OLM and its different fractions are, at least partially, mediated through downregulating melanogenic enzymes, including tyrosinase and its related protein TRP-2, and their upstream regulators MITF and phosphorylated CREB, as well as differentially upregulating ERK and JNK phosphorylations. Additionally, OLM and its fractions at concentrations used in these cellular assays did not affect cell viability of B16-F10 cells and Hs68 fibroblast cells, suggesting that such anti-melanogenic mechanisms does not involve cytotoxicity. These results demonstrated that OLM and its different fractions are promising candidates for further development as the safe and effective agent for hyperpigmentation control. The antioxidant, anti-tyrosinase, and anti-melanogenic effects of OLM and its different soluble fractions were contributed by the complicated phytoconstituents of fatty acids, steroids, phenolic compounds, tannins, and others. Further studies on the isolation and characterization of the active ingredient of OLM and its different fractions are required.

## Figures and Tables

**Table 1:** Radical-scavenging activities and mushroom tyrosinase inhibitory activities of methanolic extract of *O. laciniata* (OLM) and its different soluble fractions, including ethyl acetate (OLEF), *n*-butanol (OLBF), and water (OLWF) fractions *^a^*.

Samples	DPPH^•^ (30 min)	ABTS^+•^ (7 min)	O2^−•^ (30 min)	Tyrosinase (30 min)
	SC_50_ (µg/mL) *^c^*			IC_50_ (µg/mL) *^d^*
OLM	12.9 ± 0.1	14.2 ± 0.3	9.1 ± 0.8	97.9 ± 1.6
OLEF	12.5 ± 0.2	13.6 ± 0.2	5.8 ± 0.9	106.8 ± 2.7
OLBF	7.2 ± 0.2	10.1 ± 0.2	6.3 ± 0.2	84.8 ± 0.6
OLWF	19.3 ± 1.0	33.2 ± 0.6	18.1 ± 0.3	80.5 ± 0.9
Ascorbic acid *^b^*	5.3 ± 0.1	4.2 ± 0.3	6.5 ± 1.8	162.4 ± 11.3

*^a^* Values were expressed as the means ± SD of independent triplicates. *^b^* Ascorbic acid was used as the positive control. *^c^* SC_50_ is the concentration of test samples that scavenges 50% radicals. *^d^* IC_50_ is the concentration of test samples that inhibits 50% tyrosinase activity.

## Data Availability

Data is contained within the article or supplementary material.

## Supplementary Materials

The following are available online at https://www.mdpi.com/article/10.3390/plants10040727/s1, Figure S1: ^1^H NMR spectrum of the methanolic extract of *O. laciniata* (OLM), Figure S2: ^1^H NMR spectrum of the ethyl acetate-soluble fraction from methanolic extract of *O. laciniata* (OLEF), Figure S3: ^1^H NMR spectrum of the *n*-butanol-soluble fraction from methanolic extract of *O. laciniata* (OLBF), Figure S4: ^1^H NMR spectrum of the water-soluble fraction from methanolic extract of *O. laciniata* (OLWF).
