# Supplementary figures and images for: Oenothera laciniata Hill Extracts Exhibits Antioxidant Effects and Attenuates Melanogenesis in B16-F10 Cells via Downregulating CREB/MITF/Tyrosinase and Upregulating p-ERK and p-JNK
Source: Plants (Basel). 2021 Apr 8;10(4):727. doi: 10.3390/plants10040727 (PMC8068348; doi:10.3390/plants10040727)

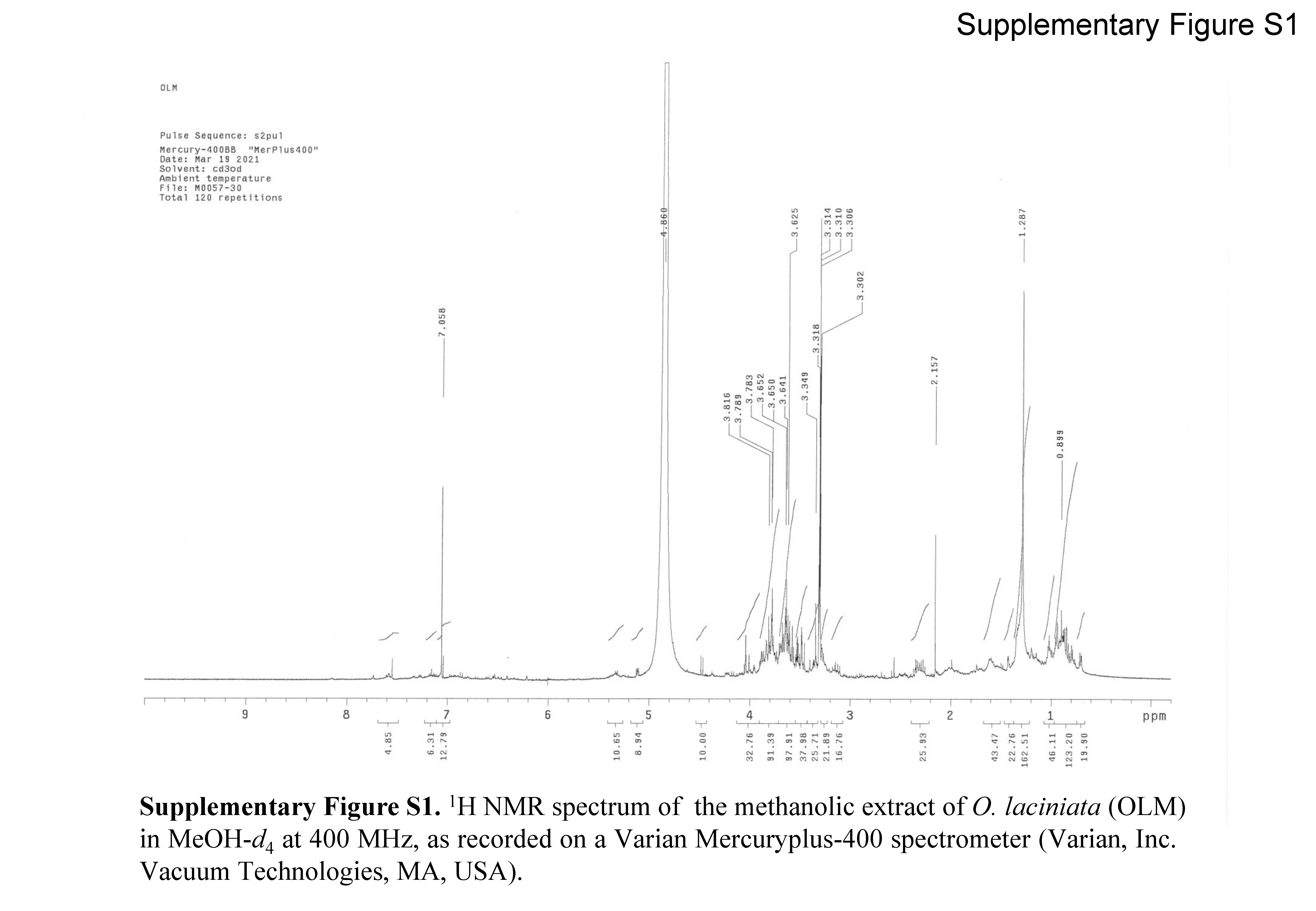

Supplement: Supplementary file 1 [file plants-10-00727-s001.zip › Oenothera laciniata_Supp Figure 1.tiff]

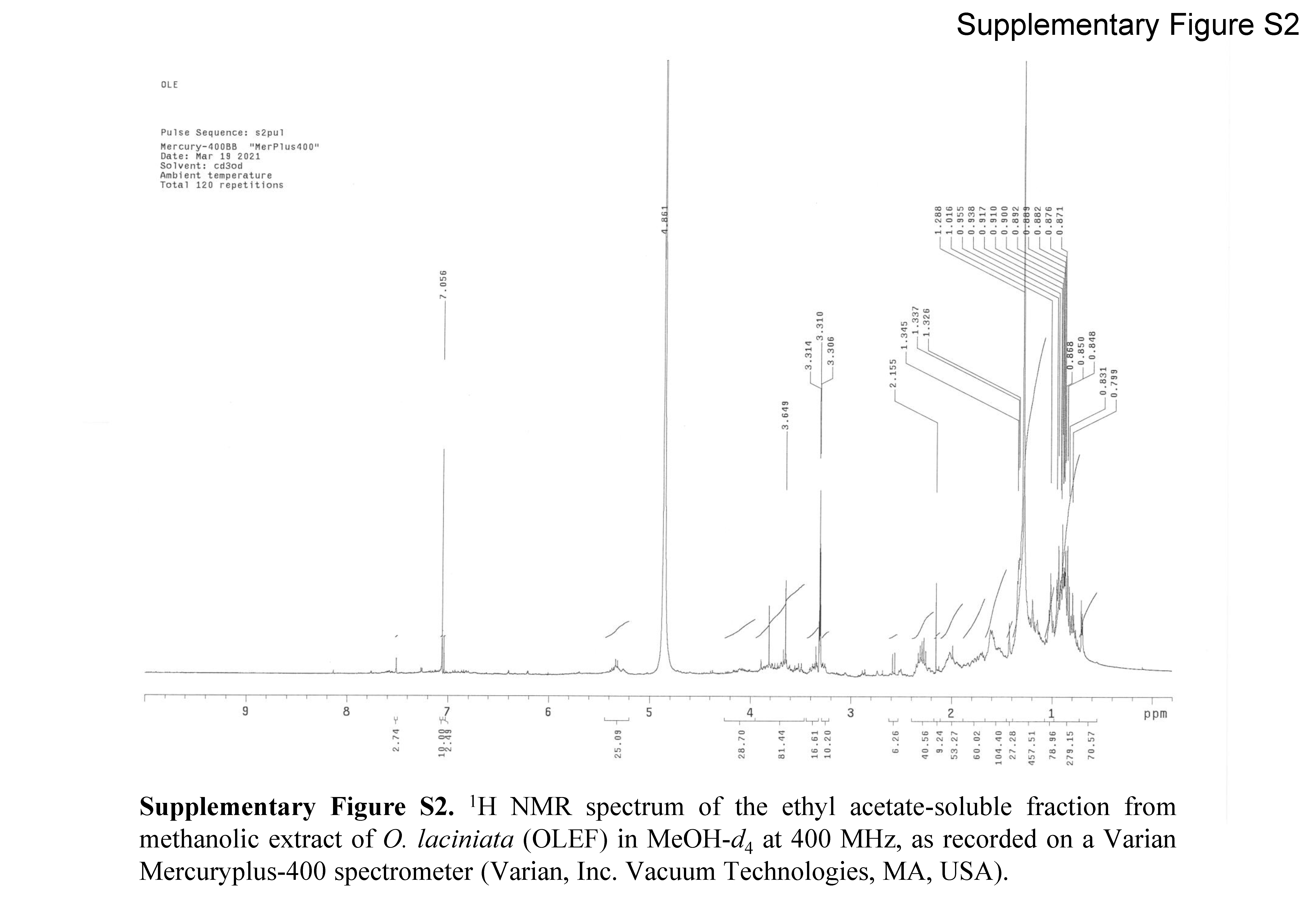

Supplement: Supplementary file 1 [file plants-10-00727-s001.zip › Oenothera laciniata_Supp Figure 2.tiff]

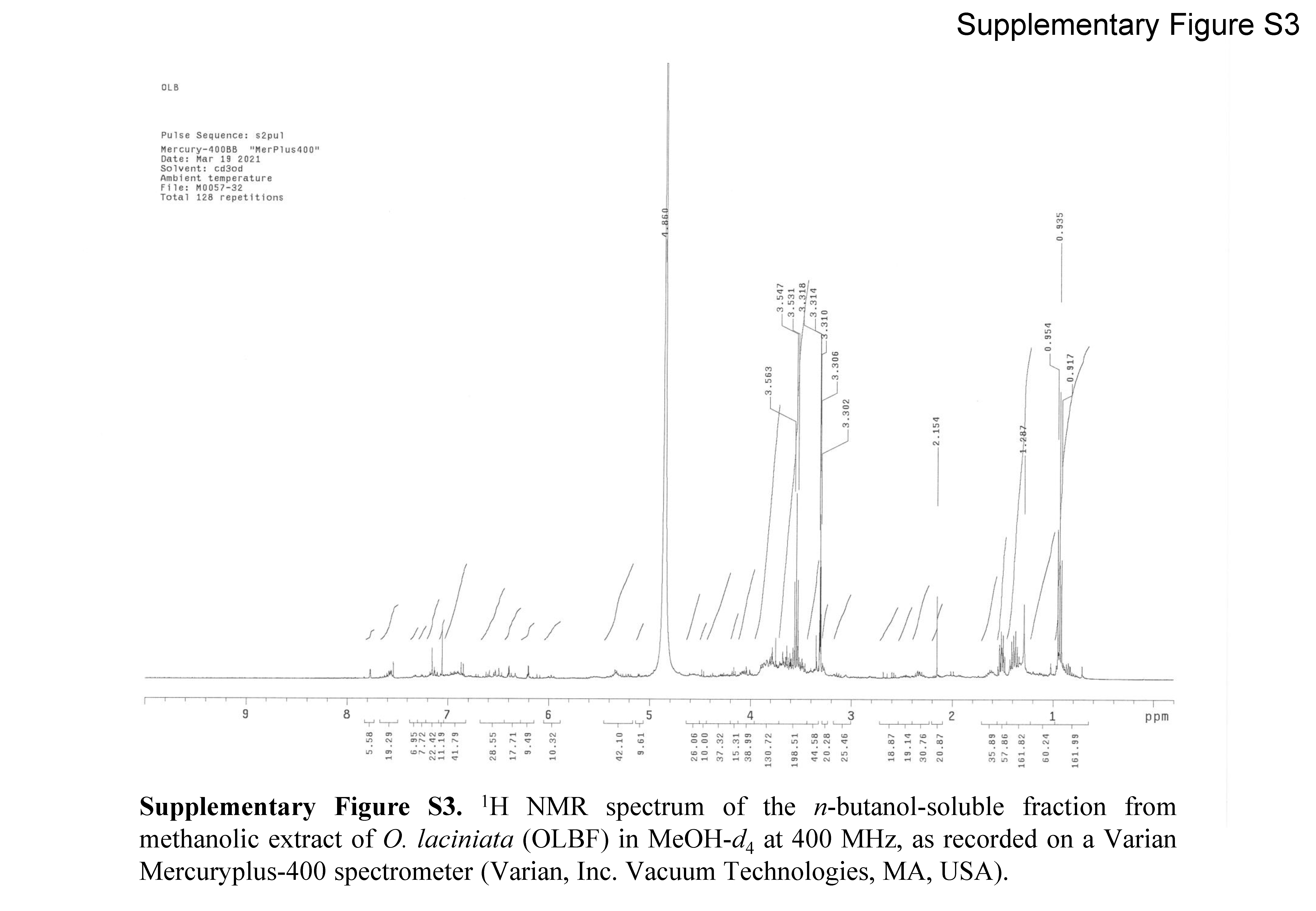

Supplement: Supplementary file 1 [file plants-10-00727-s001.zip › Oenothera laciniata_Supp Figure 3.tiff]

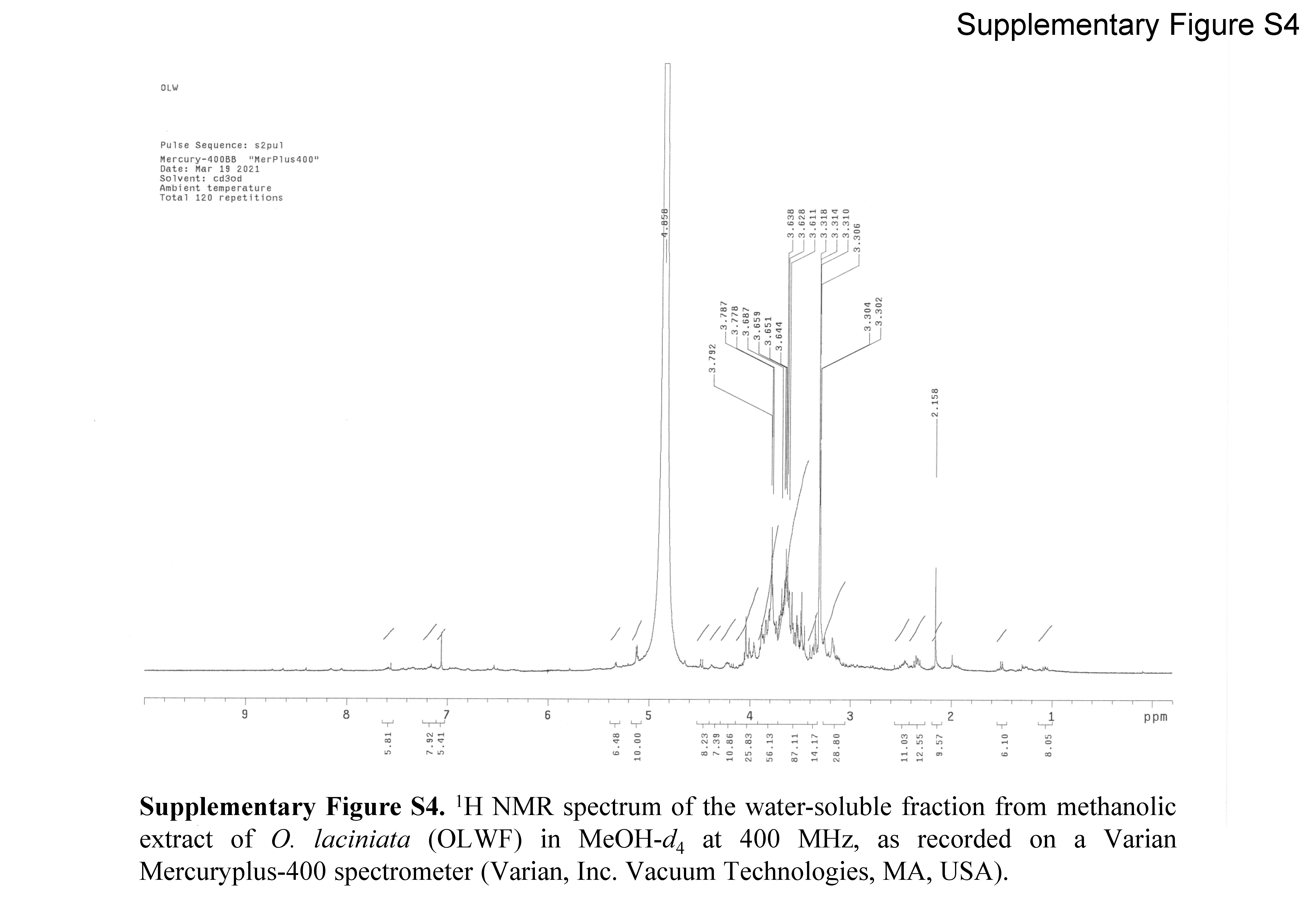

Supplement: Supplementary file 1 [file plants-10-00727-s001.zip › Oenothera laciniata_Supp Figure 4.tiff]
